# Structural Aspects of Electrospun Scaffolds Intended for Prosthetics of Blood Vessels

**DOI:** 10.3390/polym14091698

**Published:** 2022-04-21

**Authors:** Vera S. Chernonosova, Pavel P. Laktionov

**Affiliations:** 1 Institute of Chemical Biology and Fundamental Medicine, Siberian Branch, Russian Academy of Sciences, 630090 Novosibirsk, Russia; lakt@niboch.nsc.ru; 2 Meshalkin National Medical Research Center, Ministry of Health of the Russian Federation, 630055 Novosibirsk, Russia

**Keywords:** electrospinning, cell–scaffold interaction, architecture, small-diameter vascular graft, endothelial cells, blood

## Abstract

Electrospinning is a popular method used to fabricate small-diameter vascular grafts. However, the importance of structural characteristics of the scaffold determining interaction with endothelial cells and their precursors and blood cells is still not exhaustively clear. This review discusses current research on the significance and impact of scaffold architecture (fiber characteristics, porosity, and surface roughness of material) on interactions between cells and blood with the material. In addition, data about the effects of scaffold topography on cellular behaviour (adhesion, proliferation, and migration) are necessary to improve the rational design of electrospun vascular grafts with a long-term perspective.

## 1. Introduction

Cardiovascular diseases are the leading cause of disability and death worldwide, both now and as assessed for the future by experts [1,2]. The main causes of impaired blood flow in the vessel are occlusion and stenosis [3,4], demanding revascularization procedures to restore normal blood flow. Percutaneous transcatheter technologies, such as balloon angioplasty and stenting, are widely used to restore vascular patency [5]. However, in at least 30–40% of cases, these technologies are ineffective, and bypassing the defected area using vascular grafts (VGs) is demanded [6,7]. In modern surgical practice, autologous blood vessels, xenografts, or synthetic vascular grafts made of expanded poly(tetrafluoroethylene) (ePTFE) or polyethylene terephthalate (PET) is a common practice [8,9]. However, it should be noted that autologous blood vessels are rarely available [10], xenografts are prone to aneurysms [8], whereas proposed synthetic grafts are apt to stenosis [11,12] and far from desired small-diameter blood vessel (diameter less than 5 mm) substitutes in a long time perspective.

Analysis of the world market for small-diameter vascular grafts (SDVGs) demonstrates steady growth in demand for such products and especially for vascular grafts with improved biological properties fabricated from synthetic polymers [13]. These improvements must increase hemo- and biocompatibility, i.e., the ability of VG not to upset the blood hemostasis system, namely aggregation and activation of platelets, adhesion of monocytes, lysis of erythrocytes, etc. [14]. In accordance with modern concepts of biocompatibility, vascular implants should not cause inflammatory reactions and should ensure the formation of a continuous and functionally active endothelium layer on the inner surface [9,15]. The presence of such an endothelial layer is postulated as the basis for the long-term functioning of SDVG by elimination of blood contact with artificial materials and thus prime causes of thrombosis or stenosis [16]. The endothelium in SDVG borders is considered to be formed due to the proliferation of endothelial cells of an adjacent native artery, whereas a few millimeters away from anastomosis, it is formed by attaching circulating endothelial cell precursors [16,17]. In any case, endothelialization as the basis of SDVG biocompatibility depends on the potentiality of the SDVG surface to support adhesion and growth of specific cells [17,18]. Matrix recognition by cells in blood is thus the overriding stage determining the efficacy of SDVG materials. This review aimed to analyze the effect of electrospun biomaterials’ structure variation on the cells-to-surface of interactions in the bloodstream (adhesion, communication, and proliferation).

## 2. Steps of a Cell-to-Matrix Adhesion

**Figure 1:**
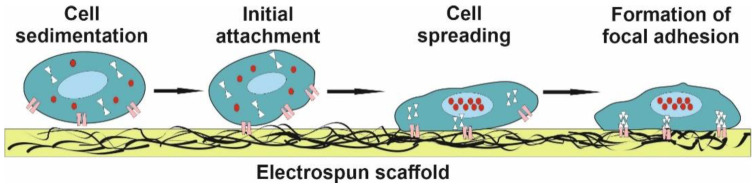
The successive stages of cell–scaffold interaction.

The primary contact of the cells with the material initiates the change of the cell shape, the induction of mechanical deformations, which leads to subsequent biochemical reactions with the generation of signals that affect cell behavior, adhesion, proliferation, migration, and differentiation [19]. There are several vital points regulating the behavior of cells in cell–material interaction (Figure 1).

At the initial stage of cell contact with the surface of the scaffolds, the cytoskeleton is deformed thanks to non-specific interactions with the surface (shearing stress) or cell adhesion molecules [20]. This interaction leads to the growth of actin filaments and the initiation of polymerization of single filaments induced by actin–myosin interactions. It should be noted that the contraction of type II myosin during the maturation of focal adhesions plays an important role in the regulation of actin filament polymerization and cell movement [21]. Integrins adhering to the surface play an essential role at the early stages of focal adhesion formation [22,23]. They indirectly interact with the actin cytoskeleton using adaptor proteins such as talin, filamin, tensin, parvin, or myosin X [24]. It is known that clustering of integrins leads to the activation of focal adhesion kinase (FAK) through autophosphorylation. The activated form of FAK kinase regulates the activity of Rac1 guanosine triphosphatase, which contributes to the improvement of actin polymerization with the formation of protrusions at the cell border and the subsequent formation of adhesion sites. FAK can also increase the activity of Rho guanosine triphosphatase, which induces the formation of stress fibers that are involved in the renewal of adhesion sites [22,25].

An increase in the number of cell contacts with the surface of materials leads to the next stage of cell-to-matrix interaction—cell adhesion. This stage is responsible for cell morphology, cell proliferation and migration, as well as the structure and mechanical integrity of the formed tissues [26]. During the formation of the cell layer on the surface of the matrices, various types of intercellular contacts are formed (gap fusion, desmosomes, tight contacts, etc.). It should be noted that receptors such as cadherins dominate in the formation of intercellular contacts [27], while integrins are mainly required for focal cell adhesion [23].

As a result of focal adhesion, cells attach to the matrix surface, and signaling between the extracellular molecules and the cytoplasm is triggered. At this stage, the characteristics of biomaterials, such as surface topography and structure, are also important since they are recognized by cell receptors that initiate signals which affect cell behavior [25,28,29,30,31]. These aspects will be discussed in the following sections of the manuscript.

## 3. The Role of Material Topography in Vascular Engineering

**Figure 2:**
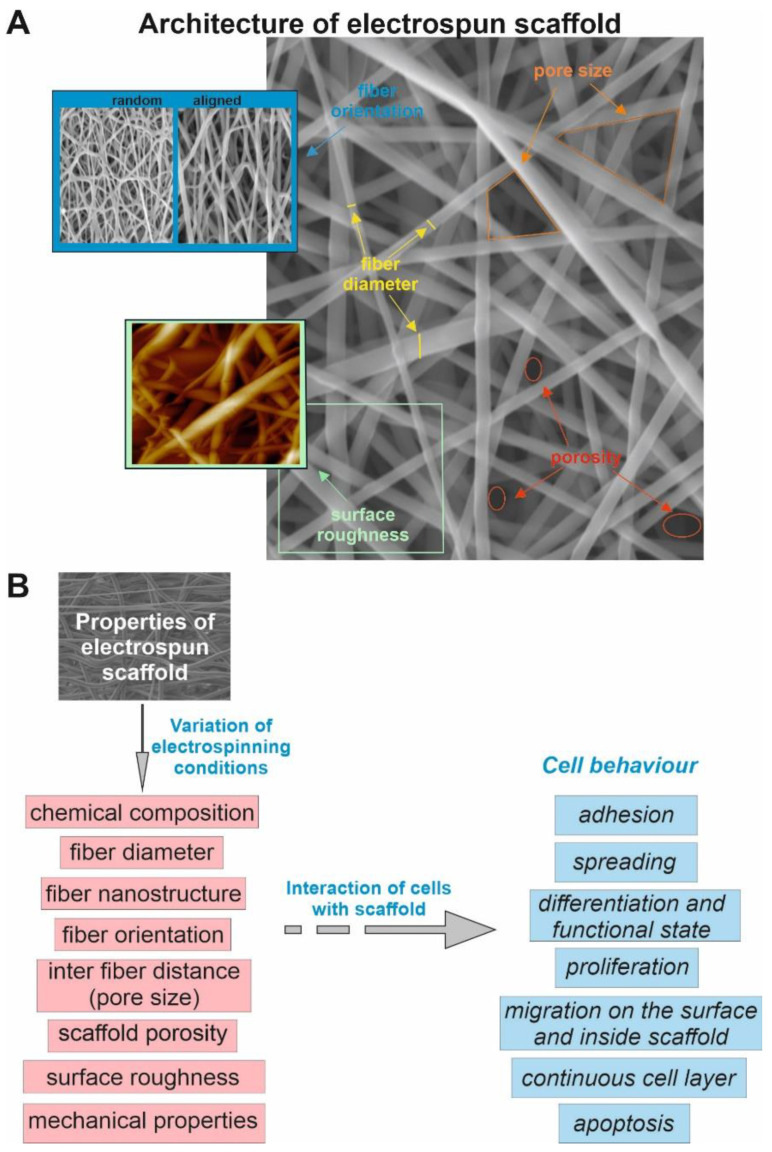
Structural parameters of electrospun scaffold (**A**). Scaffold properties affecting the behavior of cells (**B**).

Electrospinning (ES) is a widely used method for non-woven fiber production, which is used in industry, biotechnology and tissue engineering [32,33,34,35,36]. The main advantages of using the electrospinning technique for VG production are the simplicity in manufacturing fibrous scaffolds from polymer solutions and the ability to imitate the natural extracellular matrix (ECM) by producing scaffolds with native properties [37,38]. In this context, polymers with biocompatibility and good mechanical properties, including poly (ε-caprolactone), polyurethanes, poly (glycerol-co-sebacate) or poly lactic acid are widely used for fabrication of electrospun VGs [9,39,40]. It is worth mentioning that changing the electrospinning procedure parameters (the applied electric field, the rotation speed of the collector, flow rate, distance between the needle and collector, etc.) makes it possible to produce fibers ranging from a few nanometers to micrometers in diameter packed in scaffolds with different fiber orientation and structure, varied scaffold surface morphologies, and different porosity (Figure 2A) [41,42]. To characterize scaffold architecture parameters the following methods are commonly applied: Scanning electron microscopy (diameter and orientation of fibers, pore size, surface roughness, porosity), transmission electron microscopy (diameter and nanostructure of fibers), atomic force microscopy (diameter and nanostructure of fibers, surface roughness), etc. The methods used to determine scaffold characteristics were reviewed in detail by Lopez Marquez et al. [43].

Changing the surface structure of the scaffold can influence the cell–material interaction: Adhesion of the cells on the surface of the material and their ability to recognize the scaffold [30]. Figure 2B provides a resume of the important scaffold parameters affecting cellular behavior.

Data about the influence of the material architecture on cells of various origins concerning attachment, growth, and invasion are summarized in Table 1.

### 3.1. The Influence of the Surface Topography on the Cells’ Interaction with the Materials

#### 3.1.1. Fiber Diameter

Endothelialization of SDVG plays a pivotal role in maintaining their long-term function, suggesting design of surface structures that provides efficient interaction with endotheliocytes—very large cells (up to 50 µm spreading cells) and their small size precursors (around 20 µm circular cells) [66]. In particular, it was found that changes in fiber diameter can affect endothelial cell morphology, proliferation and migration (see Table 1, lines 1,2,4,5,7). In addition, it can also lead to changes in cell differentiation. 

For example, Fioretta et al. have found that variations of fiber diameter in PCL scaffold from 2 to 11 µm (2, 5, 8 and 11 µm) interfere with both endothelial colony forming cells (ECFCs) and human umbilical vein endothelial cell (HUVEC) phenotype as well as cell infiltration into the matrix [44]. Small diameter fiber (2 µm) matrices support the growth of an endothelial monolayer on the surface of the PCL matrix but not cell migration through the matrix, regardless of the cell line. The cell morphology, cytoskeleton, and expression of cell markers (CD31 and αSMA) are similar in both endothelial cell types. Cultivating ECFCs and HUVECs on matrices with 5–11 μm fibers affects cell adhesion and organization of the cellular cytoskeleton, and also promotes cell infiltration into matrices. On large-diameter fibers, ECFC cells are characterized by the alignment of actin filaments of the cytoskeleton along the axis of the fiber and the absence of intercellular contacts between ECFCs attaching to different fibers. In contrast, HUVECs are characterized by an arrangement of actin in a circle of fibers with collagen alignment in the same direction and the absence of a continuous cell layer. At the same time, it was found that the interaction of ECFC cells with 5–11 μm matrices results in the expression of α-smooth muscle actin, a characteristic marker of smooth muscle cells, which indicates the influence of the matrix structure on cell differentiation.

It should be noted that, when changing the fiber diameter, simultaneously the pore diameter and the distance between the fibers (packing density) is changed, thus creating conditions for the high rate of cell migration into matrices [58,63,67]. Moreover, it was found that pore size has a higher effect on cell migration than fiber diameter [68], but as a rule, in electrospinning, these two parameters are related to each other—matrices with lower diameter of fiber have smaller pores.

A similar effect of fiber diameter on endothelial cell lines MILE SVEN 1 and CRL-2279 proliferation and migration was also observed for biodegradable PLGA scaffolds [45]. A significant difference in the behavior of cells as a result of their interaction with the matrices consisting of 0.2 and 0.6 µm fibers was observed: At the initial period of cultivation (up to seven days), cells more effectively adhere and proliferate on the surface of a matrix with 0.2 µm fibers, without cell migration into the material. However, during the period from seven to 14 days of cell cultivation on matrices with a fiber diameter of 0.6 µm, cell proliferation increases by an average of three times due to cell infiltration compared to cells cultured on matrices with 0.2 µm fibers, preventing cell migration into matrices. At the same time, matrices of 5 μm fibers are characterized by the lowest rate of endothelial cell proliferation throughout the entire cultivation period.

It should be noted that the interaction of cells with fibers of different diameters does not affect the cellular secretion of prostacyclin [45], which can inhibit platelet activation, i.e., some cell functions are not related to the surface structure of the matrix but may be related to the chemical composition of the fiber or not depend on the properties of the matrix at all. Therefore, chemical properties of the material could be concerned with differences observed for PCL and PLGA matrixes with different fiber diameters and not only related with surface energy, contact angle, or fiber ultrastructure, but at the same time with fiber density depending on the solvent, polymer charge, and ES conditions [69].

The influence of the fiber diameter within poly(lactic-co-glycolic acid) scaffolds on the migration potential of cells and the expression of adhesive protein genes was evaluated in Ahmed’s study [47]. It has been shown that variation of fiber diameter in an interval from 0.5 to 10 µm can affect these processes. The maximum migration rate of endothelial cells 12 h after the removing of the physical barrier is observed when they are cultivated on matrices with a fiber diameter of 1 and 2 μm. After 48 h of cultivation on matrices with fiber diameters of 0.5, 1, and 2 μm, almost the entire surface of the matrix is covered with a monolayer of cells, compared with matrices with fiber diameters of 4 and 10 μm, where the cells are located in some separate areas of the surface. These data indicate that increasing the diameter of the fibers in the matrix reduces the efficiency of cell adhesion to the matrix and reduces their ability to migrate through it.

As previously mentioned, fiber diameter also affects cell morphology: On the surface of matrices with fiber diameter from 1 to 4 μm, elongated cells with a lower roundness index in the range 0.2–0.16 are observed compared with matrices with a fiber diameter of 0.5 and 10 µm (0.29 and 0.28, respectively). Using quantitative PCR, the authors studied the effect of fiber diameter in the matrix on the level of expression of secreted factors (IL-8, VEGF, and FGF) and adhesion proteins (vinculin and FAK) during the cell cultivation period on matrices with different fiber diameter. After 12 h on matrices with fiber diameters from 0.5 to 2 µm, a change in the expression of FAK tyrosine kinase, which is responsible for focal cell adhesion, was detected, while cells cultivated on matrices with different fiber diameter did not differ in the expression of the remaining five proteins [47].

#### 3.1.2. Pore Size and Material Porosity

Considering the need for continuous and functional endothelium formed on the lumen exposed surface of SDVG, not only fiber diameter but also the other structural properties of SDVG wall, like pore size and porosity, must be taken into account. Pore size influences the cells’ ability to flatten out on the matrix surface and form intercellular interactions, which in turn allows the formation of endothelial monolayer and influences the migration of endothelial cells to the inner surface of graft from the side of the anastomoses. Pore size and porosity determine the infiltration of cells into the graft wall and their viability (see Table 1, lines 14–19). Porosity ensures the exchange of nutrients and metabolites through the SDVG wall at rest and under the deformations induced by a pulse wave and stimulating the flow of fluid between pores of different sizes. It should be noted that various combinations of these parameters can affect the cell–matrix interaction, although researchers in this field do not always take these considerations into account.

Indeed, in vitro and in vivo experiments have demonstrated the influence of not only the fiber diameter, but also other parameters of the matrix. For example, it was found that such characteristics of PCL-based material such as pore size and fiber diameter affect not only cell migration, but also the polarization of macrophages [57]. Authors have shown that matrices with pore size of about 30 µm and fiber diameter of about 5–6 µm stimulate M2 macrophage polarization (pro-healing phenotype), while macrophages cultured on matrices with micropores of about 1.7 µm and fiber diameter of about 0.7 µm acquire predominantly M1 phenotype (pro-inflammatory phenotype).

A similar effect of material structure on macrophage polarization was also observed in a study by Garg et al. [58]. In this study the authors showed that the size of the matrix pores had a stronger effect on the macrophage phenotype compared to the fiber diameter. In another work, the authors found [47] that if the pore size of the matrices is smaller than the cell size, then this parameter does not affect cell adhesion and cell migration in scaffolds between matrices with fibers of different diameters.

**Figure 3:**
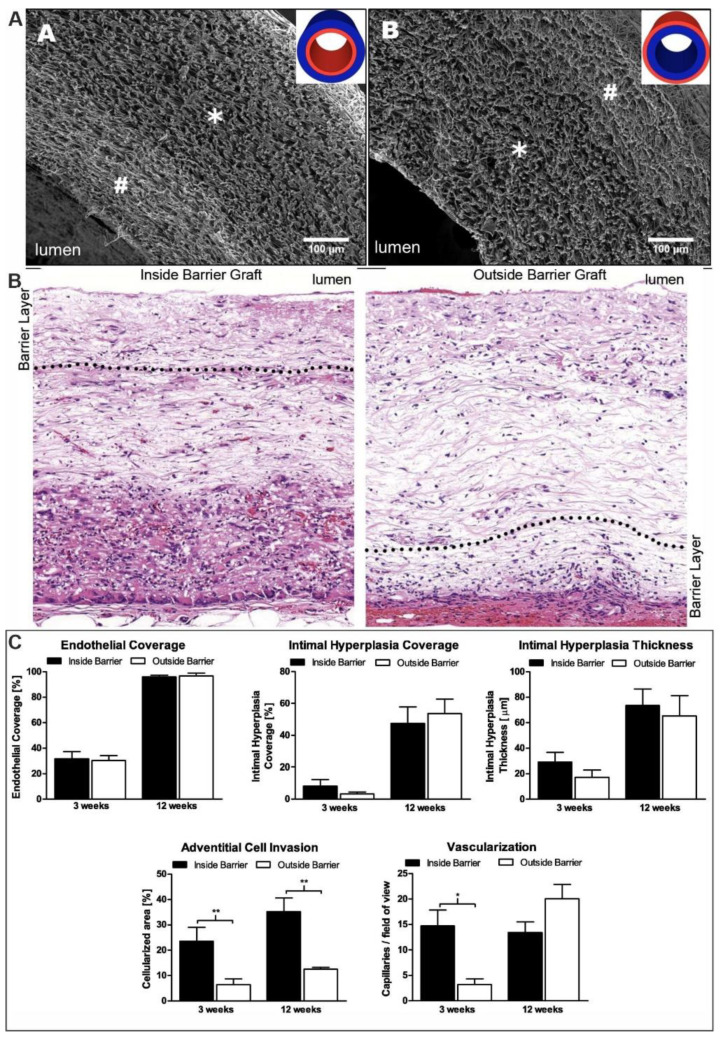
In vivo evaluation of electrospun grafts with a low-porosity layer on either the luminal or the adventitial side (reprinted from Ref. [61]. Copyright © 2012 Acta Materialia Inc. Published by Elsevier Ltd.). SEM images of electrospun vascular grafts (**A**). The low-porosity and high-porosity layers are marked as # and *, respectively. Image of H&E staining of the graft wall after 12 weeks’ implantation (**B**). Morphometric parameters of vascular grafts after 3 and 12 weeks of implantation in the rat abdominal aorta (**C**). The adventitial cell invasion is indicated by ** *p* < 0.01; vascularization is indicated by * *p* = 0.016.

The results of an in vivo study demonstrated that prostheses with macropores are characterized by an increase in cell infiltration into the prosthesis wall and increased secretion of the extracellular matrix, as well as by the vascularization process, compared with prostheses with micropores. Valence et al. investigated the role of material pore size under in vivo conditions using several types of vascular grafts as an example: PCL single layer vascular grafts produced using electrospinning with different pore sizes (9 µm or 3 µm), and PCL bilayer vascular grafts differing in the location of the slightly porous layer [61]. It was found that the pore size of the inner layer in the graft does not affect the rate of its endothelialization, the formation of the intima, and the thickness of the neointimal layer (Figure 3). Applying in vivo vascular prosthesis with a low-porosity layer on the inner side and a highly porous layer on the outer side leads to rapid neovascularization of the graft wall, which is noticeable as early as three weeks after implantation. The authors showed that placing a low-porosity layer on the outer side of the vascular graft inhibits cell migration into the prosthesis wall, suggesting that most of the cellular infiltration into the graft originates from surrounding tissues rather than from the bloodstream.

It is known that the pore size weakly correlates with the porosity of the electrospun scaffolds. For example, Garg et al. showed that increasing the pore size of a material from 1 to 10 µm leads to growth of the porosity from 69 to 81%; the porosity of the material with pore size 10 to 15 µm corresponds to 81 and 83% [58]. In another work, a twofold change in pore size increased scaffold porosity from 53 to 80% [62]. In vivo experiment showed that cells effectively migrate into the vascular wall and demonstrated their viability in a scaffold with a pore size of 4 μm and porosity 80%, compared to the scaffold with fine porosity. According to Bergmeister et al., HUVEC cells adhere and proliferate more efficiently on polyurethane matrices with high porosity (80%) compared to matrices with less porosity (53%). In vivo experiments showed that vascular grafts with high porosity are characterized by increased cell infiltration into the graft wall during the entire observation time, compared with a vascular prosthesis with a lower porosity (53%). TEM demonstrates that vascular graft with high porosity promotes the migration and growth of myofibroblasts and myocytes in the SDVG wall six months after implantation, while fragments of destroyed cells and their organelles accumulate in the wall of vascular graft with low porosity. Similar data [63] on the effect of porosity on the viability of endothelial cells were obtained for matrices with fiber diameters ranging from 5 to 12 µm and porosity from 18 to 84% produced by ES from methacrylic copolymers. It was found that the adhesion of endothelial cells to the surface of matrices does not depend on the matrix porosity and fiber orientation but depends on the conditions of cell incubation with the matrix, namely the presence of serum in the medium. After four and seven days of cells incubation with matrices, a dependence of the number of cells on the fiber orientation and the matrix porosity was observed. The most significant number of cells after seven days of incubation is observed on matrices with low porosity (18%), consisting of chaotically (non-oriented) distributed fibers, compared with matrices containing both non-oriented and oriented fibers, but having a higher porosity (35–84%). Thus, focal adhesion depends not only on the surface topography, but also on the cell culture conditions, and the surface topography plays an important role in regulating cell proliferation.

In any case, the above data suggest that the pore size can play a main or complementary role concerning the fiber diameter in the cell–matrix interaction, depending on the types of cells. Furthermore, the data of different studies on the effect of material porosity on cell migration indicate the apparent importance of this parameter for cell invasion into the material and de novo tissue formation.

#### 3.1.3. Surface Roughness of Scaffold

Fiber diameter as well as the mode of their packing in the matrix determines surface roughness [64], which can affect both cell adhesion and cell-to-cell interaction. Indeed, many studies showed that endothelial cells interact differently with matrices that differ in surface topography [70,71]. However, these data are difficult to compare and analyze, because typical roughness parameters (describing the relief of the material) like Ra (the average roughness) or Rt (the maximum profile roughness) are usually not presented.

The effect of the roughness of polyurethane 3 D-films on the adhesion and proliferation of endothelial cells was investigated by Chung [70]. The results show that growth of the surface roughness Ra from 10 to 100 nm increases adhesion and proliferation of HUVEC on the surface of film materials. In another study [64], the authors investigated the contribution of two surface parameters, fiber diameter (0.11–3.4 µm) and surface roughness (0.67–5.7 µm), to the interaction of calf pulmonary artery endothelial cells with polycaprolactone materials produced by ES. In vitro experiments showed that the maximal number of cells adhere on the surface scaffolds with Ra = 1.54 and 2.57 µm after 12 h, whereas after one to five days, the maximum proliferation of endothelial cells is observed on PCL scaffolds with a surface roughness of Ra = 2.57 μm, and 1.9 μm fiber diameter. These data are consistent with the data mentioned before, describing the effect of fiber size on the cell–matrix interaction [44].

Opposite results were obtained by other researchers [72]. It was found that HUVECs effectively adhere and proliferate on the PLGA film characterized by a roughness of 0.135 µm. The rate of cell proliferation on these both ES scaffold types having a surface roughness of 0.287 and 1.557 µm did not differ and was on average 36–40% lower compared to the film material, depending on the time of cell cultivation.

It should be noted that the surface roughness is an equivalent structural parameter, as is the diameter of the fibers, since it determinates a number of cell–matrix contacts at the matrix recognition stage and further. The optimal value of the surface roughness of electrospun matrices for endotheliocytes is challenging to define due to many studies’ lack of roughness characteristics. However, in summary, the data that presented the scaffolds with a roughness range up to 1.5 µm represent a good support for at least the proliferation of endothelial cell.

#### 3.1.4. Fiber Orientation

A number of studies [49,50,51,52,53,54,55] have shown that cell morphology and migration are affected not only by fiber diameter, but also by their orientation in the scaffold. Indeed, it is suggested that the fiber orientation in the matrix affects the initial stage of cell adhesion, which further determines the strength of the interaction of the cellular layer with the matrix. Naturally, as the study’s results show, fiber orientation is not the only parameter that determines cell adhesion and growth.

**Figure 4:**
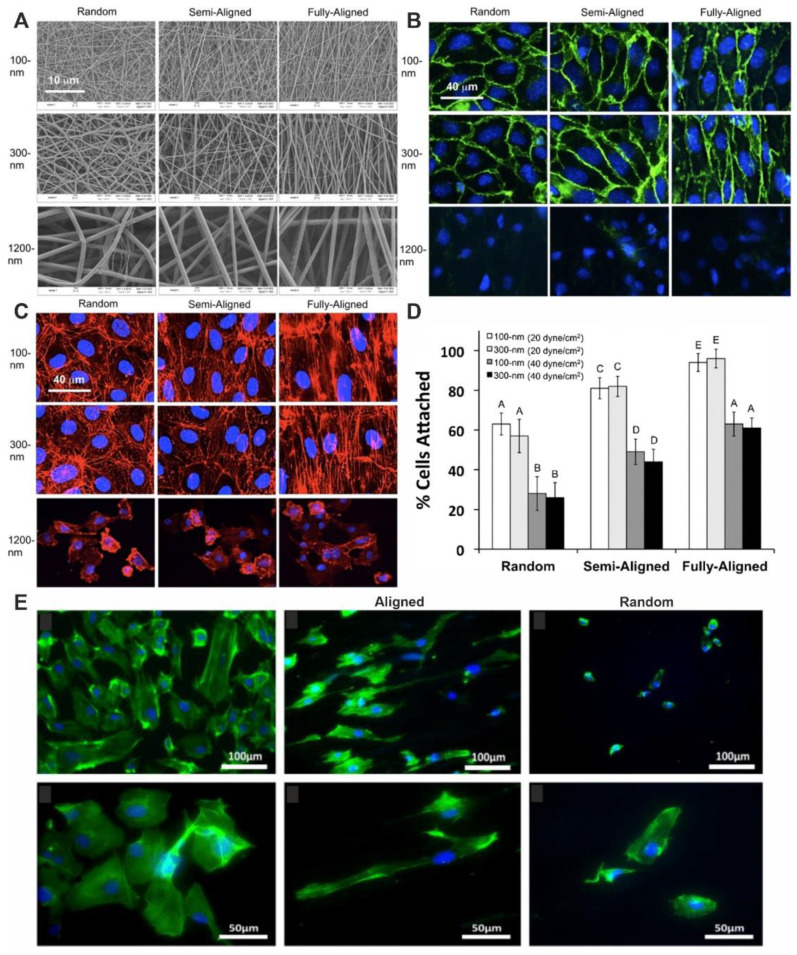
The effect of the scaffold’s fiber orientation on HUVEC morphology and alignment on electrospun scaffolds. The influence of fiber orientation and medium flow on interaction of endothelial cells with scaffolds (reprinted from Ref. [49]. Copyright © 2013 Biotechnology and Bioengineering Inc. Published by Wiley Periodicals). SEM image of electrospun PCL/collagen scaffolds (**A**). Fluorescent images of cytoskeletal F-actin organization (**B**) and VE-cadherin expression (**C**) for endothelial cells on scaffolds with varying fiber diameters and orientations. The number of cells that adhered to materials after 60 min of continuous hydrodynamic shear stress (**D**). Image (**E**) was obtained by fluorescent microscopy (cells were cultured with different scaffolds for five days and stained with Alexa Fluor 488 Phalloidin and DAPI) (reprinted from Ref. [51]. Copyright © 2011 Acta Materialia Inc. Published by Elsevier Ltd.)

The influence of fiber diameter (0.1, 0.3 and 1.2 µm), and the orientation (chaotic, partially oriented and fully oriented, see Figure 4A) on the interaction of HUVEC endothelial cells with matrices from a mixture of PCL and type I collagen was studied [49]. The authors found that cells cultured on matrices with oriented fibers 0.1 and 0.3 µm in diameter have an elongated shape and are characterized by the parallel location of actin fibers with the matrix fibers, with intense expression of VE-cadherin at the cell periphery at the points of contact between cells and fibrous material (Figure 4B,C). For cells cultured on matrices with a fiber diameter of 1.2 µm, no effect of the orientation of matrix fibers on cell morphology was found, and no overexpression of VE-cadherin in cells was observed. The endothelial cell monolayer is formed on the surface of matrices with 0.1–0.3 µm fiber diameter, while on the surface of matrices with 1.2 µm fiber diameter; the continuous monolayer is not formed, and cells tend to migrate into the matrix. It should be noted that, in contrast to the static conditions of cell cultivation, under dynamic conditions, i.e., in a fluid flow at a shear of 20–40 dyn/cm^2^, cell adhesion depends on the orientation of the fibers, and practically does not depend on the fiber diameter (Figure 4D). At a shear of 20 dyn/cm^2^, 95% of the cells adhere to the matrices with oriented fibers along the flow, but only 80% and 60% of the cells adhere to the surface of the matrices with partially oriented and randomly directed fibers, respectively. At a shear of 40 dyn/cm^2^, 62% of cells adhere to the surface of matrices with oriented fibers, while 46% and 27% of cells adhere to the surface of matrices with partially oriented and chaotically distributed fibers, respectively. These results demonstrate that endothelial cells attach more efficiently to matrices with flow-oriented fibers, and they support the formation of a continuous endothelium; thus, matrices with flow-oriented fibers are better candidates for vascular graft tissue engineering.

Similar results were described in other works [50,51]. The authors compared the interaction of HUVECs with PCL scaffolds consisting of randomized and oriented microfibers and nanofibers [50]. On the surface of matrices with oriented fibers with a diameter of 0.3 and 1.3 µm, cells proliferate during the first five days of cultivation much faster compared to matrices with random fibers (0.4 and 2.4 µm diameters), have a spindle shape, and are oriented along the matrix fibers. It should be noted that cells cultured on 0.3 µm oriented fibers remain oriented along the fibers even at later times of cultivation. Concerning the effect of fiber orientation on the morphology, orientation, and proliferation of HUVECs, it was found that on oriented-fiber matrices produced by ES the cells (Figure 4E) are distributed along the fibers’ orientation and have a spindle shape [51]. Cells seeded on matrices with random fiber orientation did not obtain this shape (Figure 4E). Thus, the authors demonstrate that changes in cells under the influence of surface topography of the matrix occur as a result of cell adhesion to the matrix, namely, cell elongation, alignment, and organization of F-actin filaments indicate the importance of cell–matrix interaction. To be fair, it is worth remembering that the chemical composition of the fibers is also important and the patterns observed may not be so pronounced on other polymers, as well as when fiber fusion occurs.

Summarizing the data on the effect of material structure parameters (fiber size and orientation, pore size, and porosity) on the processes determined by cell–material interaction demonstrates that scaffolds with a fiber diameter of 0.1 to 0.5 µm are well suited for SDVG as their surface is well compatible with endothelialization. As a result of the contact of endothelial cells such scaffold, effective adhesion, and proliferation of endothelial cells are observed with the formation of a cellular monolayer on the surface of the material, the ability of cells to migrate is preserved, and, in general, there is no visible change in the phenotype of endothelial cells. Using matrices with fiber diameter more than 1.5 μm, the cell division rate, their ability to fill the surface and form a continuous layer, decreases, and endothelial cells show tend to change into not typical phenotype. The unidirectional orientation of the fibers in the matrix promotes endothelial cell adhesion and the formation of a continuous endothelial layer. There are scaffolds with pores smaller than endothelial cell size, low porosity, and roughness apt to be endothelized. It should be noted that matrices with 0.1 µm fibers do not meet the most important requirements for prostheses, namely their strength; therefore scaffolds with about 1 µm fibers represent compromised solution for production of SDVG.

### 3.2. The Influence of the Material Topography on the Interaction with Blood Cells

Implantation of SDVG suggests its immediate contact with blood plasma and blood cells; thus, hemocompatibility is an urgent demand. The primary source of early stenosis is the activation of the blood coagulation system and thrombus formation due to the interaction of blood cells with the scaffold surface. An analysis of the literature data shows that the effect of the structural parameters of materials on the blood cell–scaffold interaction is not studied enough. This section summarizes data on the interconnection between structural parameters of materials and their blood-compatible properties (see Table 1, lines 3,5,13,17).

It was found that the fiber diameter has a fundamental influence on the material hemocompatibility as a result of direct contact with human blood, rather than the chemical composition of the material. It was shown that an increase in fiber diameter in the electrospun matrix leads to an increase in surface roughness and porosity of the material. The use of matrices with fiber diameter smaller than 1 µm causes less activation of the coagulation cascade and less platelet adhesion than matrices consisting of larger fibers. Platelets adhere more effectively to materials consisting of fibers with a diameter of 1–2 μm, leading to thrombin formation on their surface. At the same time, the highest adhesion and activation of platelets and activation of the coagulation cascade are observed when using electrospun materials with fiber diameter more than 5 microns. These results are consistent with other data obtained in the study of other types of surface topography (pillars, struts [73,74,75]) showing that platelet adhesion and blood reaction depend on surface roughness [76,77].

Platelet adhesion is determined by the ability to adhere to scaffolds and penetrate deep into the material. The small size of platelets allows them to form multiple contacts only with scaffolds consisting of densely packed fibers of small diameter or with very large fibers and pores. This suggestion was confirmed in reference [48], demonstrating that an increase in fiber diameter in ES that produces PCL scaffolds from 0.07 to 3.4 µm leads to a decrease in platelet adhesion. The maximum adhesion of platelets is observed on the surface of scaffolds with 0.07 µm fibers and amounts to 1.13 × 10^6^ platelets/cm^2^; for fibers with diameter of 1.45 µm or more, the number of adherent platelets is no more than 0.32 × 10^6^ platelets/cm^2^. In the same study it was found that erythrocytes (7.6 × 2 μm size) are effectively retained in the pores of the material upon contact with matrices with a fiber diameter of more than 1.45 µm, which leads to an increase in the level of hemolysis, compared with scaffold containing fibers of a smaller diameter. The scaffolds with a diameter of fibers ranging from 0.58 to 1.45 µm demonstrate the best anticoagulant properties. Unfortunately, the study did not provide data on scaffold porosity and surface roughness, which can be directly interconnected with blood cell–scaffold interactions. It is logical to assume that low-porosity materials are suitable for developing blood contacting devices, since blood cells will enter the material in a minimum level with a minimal level of hemolysis and platelet activation.

The results of the following work confirm the above hypothesis. In vivo study of two-layer PCL electrospun grafts contained a low-porosity layer (pore size of 3 μm and a porosity of 60%) with localization inside or outside the graft lumen revealed a two- and six-fold decrease in hemolysis, compared with the prosthesis-containing layer with pore size of 9 μm and a porosity of 80%. The harm from blood cells that penetrated the SDVG wall was confirmed in the study [62], demonstrating that impregnation of blood cells in the wall of highly porous polyurethane vascular grafts (pore size about 4 μm and porosity 80%) during implantation may promote parietal thrombosis. Nevertheless, according to histological studies of explanted SDVG, they were not characterized by increased thrombus formation and thus were assumed as suitable for vascular graft engineering.

**Figure 5:**
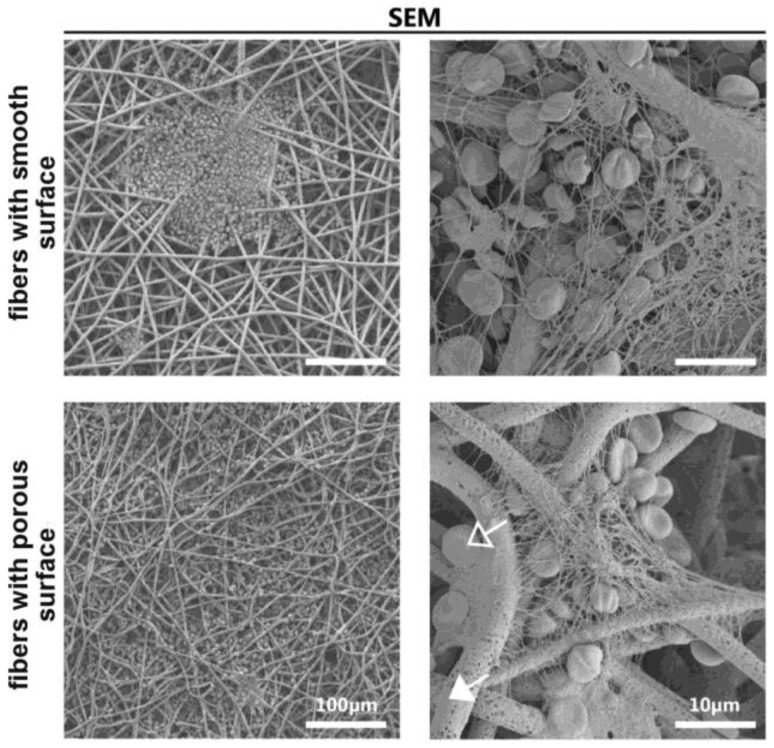
SEM images were obtained after 1 h whole blood incubation on electrospun scaffolds containing fibers with smooth or porous surface (reprinted from Ref. [56]. Copyright © 2018 Biointerphases Inc. Published by the American Vacuum Society.)

The nano porosity of the fibers, arising due to solvent evaporation or phase separation, in principle can contribute to the cell-to-matrix interaction. However, the reliable effects of fiber structure (smooth or porous surface) on the interaction of scaffolds with blood cells were well described. In a single study it was found that the scaffolds containing both porous fibers and smooth fibers have similar blood clotting values, depending on the concentration of platelet factor-4 in supernatants (about 400 ng depending on the blood donor) [56]. SEM results have shown that the number of adherent platelets and erythrocytes, as well as the area with formation of fibrin on the materials surface (Figure 5), does not depend on the structure of the fiber.

## 4. Conclusions

Structural properties of the surface of the scaffold are directly related to the formation of cell contacts with the surface and cell adhesion, spreading, and proliferation. Therefore, to form a cell monolayer, scaffold characteristics such as fiber diameter, fiber packing density, porosity, and surface roughness are essential since they affect the possibility of forming cell–scaffold contacts and the cell-to-cell interactions.

Considering the application as mentioned earlier of the materials, the following structural characteristics of the materials are to be profitable:

− surface roughness of biomaterials on a scale of 50–300 nm;
− fiber diameter in the range 0.5–1 µm;
− pore size in the range 3–7 µm;
− porosity of electrospun scaffold about 35–80%.

It should be mentioned that these material characteristics are obtained at static conditions. The blood pulse flow, shearing stress, and blood pressure could interfere with fiber diameter and orientation, surface roughness, porosity, and thus cell-to-matrix interactions. At the same time, long-term processes must be investigated together with the earlier suggestion to reveal the importance of the individual surface characteristics of electrospun scaffold and reveal the optimal material for SDVG fabrication.

## Figures and Tables

**Table 1:** Characteristics of electrospun scaffolds and cell-to-matrix interaction in vitro/in vivo.

	Polymer	Structural Characteristics of Electrospun Scaffolds						Model In Vitro/ In Vivo	Effects	Ref
		Fiber Diameter, µm	Pore Size, µm	Fiber Orientation	Structure of Fibers	Porosity (%)	Surface Roughness			
1	PCL	2; 5; 8; 11	11.5; 50.4; 76.2; 116.1					ECFCs; HUVECs	− pore size promoted of cell infiltration into material − fiber size influenced on cell phenotype and cell behavior	[44]
2	PLGA	0.2; 0.6; 1.5; 5	1.4; 8.5; 10.9; 27.3			80.9; 85.2; 87.3; 89.8		MILE SVEN 1; CRL-2279	− materials with fiber diameter 0.6 µm demonstrated homogeneously proliferate of endothelial cells − fiber size not effected on secretion of prostacyclin	[45]
3	Degrapol; PLGA	<1; 2–3; >5	33.9–43; 226–368; 988–1623				1.46–1.5; 3.9–3.94; 5–6.26	blood	− increase of fiber diameter stimulated platelet adhesion and activation − scaffold morphology is main than chemistry of the polymers	[46]
4	PLGA	0.5; 1; 2; 4; 10		aligned				HUVECs	− fiber size induced changes in cell shape and orientation, and control migration velocity − maximum expression of tyrosine kinase associated with focal adhesion for materials with 0.5–2 µm fibers	[47]
5	PCL	0.07; 0.58; 1.45; 2.04; 2.74; 3.44		aligned				HUVECs; blood	− fiber size affected the platelet adhesion on the material surface (maximum adhesion for 0.07 µm fibers, minimum—for fiber ≥2 µm − fiber size induced changes in cytoskeleton of HUVECs and has not effect cell adhesion and proliferation	[48]
6	PCL with type I collagen	0.1; 0.3; 1.2		random; semi-aligned; aligned				HUVECs	− fiber orientation determined morphological and structural changes (cell elongation, alignment, and F-actin organization) of HUVECs	[49]
7	PCL	0.3–0.4; 1.3–2.4		random; aligned				HUVECs	− materials with aligned fibers and diameter 0.3–0.4 µm significantly enhanced cellular proliferation	[50]
8	Gelatin with fibroblast growth factor	0.83–1		random; aligned				HUVECs	− fiber orientation determined cell morphology and alignment	[51]
9	PCL; PCL with gelatin	0.75; 0.65		random; aligned		86; 73		Human mesenchymal stem cells	− fiber orientation had a pronounced effect on cell morphology and orientation	[52]
10	Copolyetheresterurethane	0.5; 2		random; aligned				HUVECs; SMCs	− fiber orientation increases adhesion and proliferation of HUVECs, but reduces cell viability of both type cells − materials with fibers 2 µm increases HUVECs proliferation and cell viability, but reduces SMCs viability	[53]
11	PCL; PCL with gelatin	0.25; 0.4		random; aligned				rabbit cardiomyo-cytes	− fiber orientation had a pronounced effect on cell morphology	[54]
12	Poly(vinyl alcohol); poly(vinyl alcohol) with gelatin	0.11		random; aligned				mouse fibroblast (3T3)	− fiber orientation had a pronounced effect on cell morphology, had not effect cell proliferation	[55]
13	PCL	3.6–3.7			porous fibers; smooth fibers			HUVECs, blood	− fiber surface structure had not effect on adhesion and proliferation of HUVECs, adhesion of platelets and fibrinogen adsorption.	[56]
14	PCL	0.7; 5.6	4.6; 41			66; 83		macrophages RAW264.7	− macrophages cultured on micro-fiber scaffolds tended to polarize into the immunomodulatory and tissue remodeling phenotype (M2)	[57]
								rat AA	− PCL grafts with larger pores and microfibers markedly enhanced cell infiltration, vascularization and efficient regeneration of functional tunica media in comparison with the PCL grafts with nanofibers.	
15	Polydioxanone	0.35; 2.2; 2.8	1; 10.5; 15			69; 81; 83		mouse bone marrow- derived macrophages	− pore size of the scaffold had a stronger effect on the macrophage phenotype compared to the fiber diameter − correlation has been shown between pore size and expression of markers of the regenerative (arginase 1)/inflammatory (inducible nitric oxide synthase) phenotype, as well as the secretion of angiogenic cytokines (VEGF, TGF-β1 and bFGF)	[58]
16	PCL	0.4; 2.7	0.1–1.8; 0.4–0.6; 8–10; 44–64					HUVECs	− maximum rate of cell adhesion and proliferation was observed for scaffold with fibers of 2.7 μm and a pore size of ~44–64 μm − pore size promoted cell proliferation and infiltration into materials regardless of fiber diameter	[59,60]
17	PCL	0.8; 2.2	3; 9			60; 80		rat AA	− graft with 80% porosity had the highest blood leakage rate (1.55 mL × cm^–2^ × min^-1^). − pore size of scaffold did not affect the rate of endothelialization, the formation and thickness of the intima layer	[61]
18	Polyurethane	0.9; 1	2; 4			53; 80		HUVECs	− HUVECs effectively adhered to the surface of scaffold with 80 % porosity	[62]
								rat AA	− Grafts with a pore size of 4 µm and 80% porosity had efficiently cell migration and proliferation	
19	Hexyl methacrylate, methyl methacrylate and methacrylic acid	5–12		random; aligned		18–84		HUVECs	− proliferation HUVECs depended more on the scaffold porosity than on the fiber orientation in the scaffold	[63]
20	PCL	0.11; 0.8; 1.9; 3.4					0.67;1.47; 2.57;5.7	calf pulmonary artery endothelial cell line	− maximum adhesion and proliferation of endothelial cells was observed at Ra = 2.57 μm	[64]
21	PLLA	0.23; 3.5					0.28; 1.5	HUVECs	− surface roughness had affect cell behavior	[65]

PCL—polycaprolactone; PLGA—poly(lactic-co-glycolic acid); PLLA—poly(L-lactic acid); HUVECs—umbilical vein endothelial cells; SMCs—smooth muscle cells; rat AA—rat abdominal aorta; Ra—roughness.

## Data Availability

The data presented in this study are available on request from the corresponding author.

## References

[1] Amini M., Zayeri F., Salehi M. (2021). Trend analysis of cardiovascular disease mortality, incidence, and mortality-to-incidence ratio: Results from global burden of disease study 2017. BMC Public Health.

[2] Roth G.A., Mensah G.A., Johnson C.O., Addolorato G., Ammirati E., Baddour L.M., Barengo N.C., Beaton A.Z., Benjamin E.J., Benziger C.P. (2020). Global Burden of Cardiovascular Diseases and Risk Factors, 1990–2019: Update From the GBD 2019 Study. J. Am. Coll. Cardiol..

[3] Pashneh-Tala S., MacNeil S., Claeyssens F. (2016). The Tissue-Engineered Vascular Graft-Past, Present, and Future. Tissue Eng. Part B Rev..

[4] Herrington W., Lacey B., Sherliker P., Armitage J., Lewington S. (2016). Epidemiology of Atherosclerosis and the Potential to Reduce the Global Burden of Atherothrombotic Disease. Circ. Res..

[5] Aboyans V., Ricco J.-B., Bartelink M.-L.E.L., Björck M., Brodmann M., Cohnert T., Collet J.-P., Czerny M., de Carlo M., Debus S. (2018). 2017 ESC Guidelines on the Diagnosis and Treatment of Peripheral Arterial Diseases, in collaboration with the European Society for Vascular Surgery (ESVS): Document covering atherosclerotic disease of extracranial carotid and vertebral, mesenteric, renal, upper and lower extremity arteriesEndorsed by: The European Stroke Organization (ESO)The Task Force for the Diagnosis and Treatment of Peripheral Arterial Diseases of the European Society of Cardiology (ESC) and of the European Society for Vascular Surgery (ESVS). Eur. Heart J..

[6] Lin Y., Tang X., Fu W., Kovach R., George J.C., Guo D. (2015). Stent fractures after superficial femoral artery stenting: Risk factors and impact on patency. J. Endovasc. Ther..

[7] Laird J.R., Katzen B.T., Scheinert D., Lammer J., Carpenter J., Buchbinder M., Dave R., Ansel G., Lansky A., Cristea E. (2012). Nitinol stent implantation vs. balloon angioplasty for lesions in the superficial femoral and proximal popliteal arteries of patients with claudication: Three-year follow-up from the RESILIENT randomized trial. J. Endovasc. Ther..

[8] Chlupáč J., Filová E., Bačáková L. (2009). Blood vessel replacement: 50 years of development and tissue engineering paradigms in vascular surgery. Physiol. Res..

[9] Leal B.B.J., Wakabayashi N., Oyama K., Kamiya H., Braghirolli D.I., Pranke P. (2020). Vascular Tissue Engineering: Polymers and Methodologies for Small Caliber Vascular Grafts. Front. Cardiovasc. Med..

[10] Isenberg B.C., Williams C., Tranquillo R.T. (2006). Small-diameter artificial arteries engineered in vitro. Circ. Res..

[11] Zilla P., Bezuidenhout D., Human P. (2007). Prosthetic vascular grafts: Wrong models, wrong questions and no healing. Biomaterials.

[12] Drews J.D., Miyachi H., Shinoka T. (2017). Tissue-engineered vascular grafts for congenital cardiac disease: Clinical experience and current status. Trends Cardiovasc. Med..

[13] Vascular Grafts Market Size, Share & Trends Analysis Report by Product, by Application (Cardiac Aneurysm, Kidney Failure, Coronary Artery Disease, by Raw Material (Polyester, Polyurethane), by Region, and Segment Forecasts, 2021–2028. https://www.grandviewresearch.com/industry-analysis/vascular-graft-market.

[14] Radke D., Jia W., Sharma D., Fena K., Wang G., Goldman J., Zhao F. (2018). Tissue Engineering at the Blood-Contacting Surface: A Review of Challenges and Strategies in Vascular Graft Development. Adv. Healthc. Mater..

[15] Benrashid E., McCoy C.C., Youngwirth L.M., Kim J., Manson R.J., Otto J.C., Lawson J.H. (2016). Tissue engineered vascular grafts: Origins, development, and current strategies for clinical application. Methods.

[16] Sánchez P.F., Brey E.M., Briceño J.C. (2018). Endothelialization mechanisms in vascular grafts. J. Tissue Eng. Regen. Med..

[17] Melchiorri A.J., Hibino N., Fisher J.P. (2013). Strategies and techniques to enhance the in situ endothelialization of small-diameter biodegradable polymeric vascular grafts. Tissue Eng. Part B Rev..

[18] Zhuang Y., Zhang C., Cheng M., Huang J., Liu Q., Yuan G., Lin K., Yu H. (2021). Challenges and strategies for in situ endothelialization and long-term lumen patency of vascular grafts. Bioact. Mater..

[19] Geiger B., Bershadsky A., Pankov R., Yamada K.M. (2001). Transmembrane crosstalk between the extracellular matrix--cytoskeleton crosstalk. Nat. Rev. Mol. Cell Biol..

[20] Huber F., Schnauß J., Rönicke S., Rauch P., Müller K., Fütterer C., Käs J. (2013). Emergent complexity of the cytoskeleton: From single filaments to tissue. Adv. Phys..

[21] Svitkina T. (2018). The Actin Cytoskeleton and Actin-Based Motility. Cold Spring Harb. Perspect. Biol..

[22] Wozniak M.A., Modzelewska K., Kwong L., Keely P.J. (2004). Focal adhesion regulation of cell behavior. Biochim. Biophys. Acta.

[23] Kanchanawong P., Shtengel G., Pasapera A.M., Ramko E.B., Davidson M.W., Hess H.F., Waterman C.M. (2010). Nanoscale architecture of integrin-based cell adhesions. Nature.

[24] Zaidel-Bar R., Itzkovitz S., Ma’ayan A., Iyengar R., Geiger B. (2007). Functional atlas of the integrin adhesome. Nat. Cell Biol..

[25] Roca-Cusachs P., Iskratsch T., Sheetz M.P. (2012). Finding the weakest link: Exploring integrin-mediated mechanical molecular pathways. J. Cell Sci..

[26] Parsons J.T., Horwitz A.R., Schwartz M.A. (2010). Cell adhesion: Integrating cytoskeletal dynamics and cellular tension. Nat. Rev. Mol. Cell Biol..

[27] Michael M., Yap A.S. (2013). The regulation and functional impact of actin assembly at cadherin cell-cell adhesions. Semin. Cell Dev. Biol..

[28] Zhang J., Ma X., Lin D., Shi H., Yuan Y., Tang W., Zhou H., Guo H., Qian J., Liu C. (2015). Magnesium modification of a calcium phosphate cement alters bone marrow stromal cell behavior via an integrin-mediated mechanism. Biomaterials.

[29] Zhu M., Wang Z., Zhang J., Wang L., Yang X., Chen J., Fan G., Ji S., Xing C., Wang K. (2015). Circumferentially aligned fibers guided functional neoartery regeneration in vivo. Biomaterials.

[30] Li Y., Xiao Y., Liu C. (2017). The Horizon of Materiobiology: A Perspective on Material-Guided Cell Behaviors and Tissue Engineering. Chem. Rev..

[31] Marino A., Genchi G.G., Sinibaldi E., Ciofani G. (2017). Piezoelectric Effects of Materials on Bio-Interfaces. ACS Appl. Mater. Interfaces.

[32] Fadil F., Affandi N.D.N., Misnon M.I., Bonnia N.N., Harun A.M., Alam M.K. (2021). Review on Electrospun Nanofiber-Applied Products. Polymers.

[33] Hanumantharao S.N., Rao S. (2019). Multi-Functional Electrospun Nanofibers from Polymer Blends for Scaffold Tissue Engineering. Fibers.

[34] Liu Z., Ramakrishna S., Liu X. (2020). Electrospinning and emerging healthcare and medicine possibilities. APL Bioeng..

[35] Salmeri M., Ognibene G., Saitta L., Lombardo C., Genovese C., Barcellona M., D’Urso A., Spitaleri L., Blanco I., Cicala G. (2020). Optimization of ZnO Nanorods Growth on Polyetheresulfone Electrospun Mats to Promote Antibacterial Properties. Molecules.

[36] Su Z., Dinga J., Wei G. (2014). Electrospinning: A facile technique for fabricating polymeric nanofibers doped with carbon nanotubes and metallic nanoparticles for sensor applications. RSC Adv..

[37] Khorshidi S., Solouk A., Mirzadeh H., Mazinani S., Lagaron J.M., Sharifi S., Ramakrishna S. (2016). A review of key challenges of electrospun scaffolds for tissue-engineering applications. J. Tissue Eng. Regen. Med..

[38] Gao J., Jiang L., Liang Q., Shi J., Hou D., Tang D., Chen S., Kong D., Wang S. (2018). The grafts modified by heparinization and catalytic nitric oxide generation used for vascular implantation in rats. Regen. Biomater..

[39] Zhang Q., Bosch-Rué È., Pérez R.A., Truskey G.A. (2021). Biofabrication of tissue engineering vascular systems. APL Bioeng..

[40] Rickel A.P., Deng X., Engebretson D., Hong Z. (2021). Electrospun nanofiber scaffold for vascular tissue engineering. Mater. Sci. Eng. C Mater. Biol. Appl..

[41] Castaño O., Eltohamy M., Kim H.-W. (2012). Electrospinning technology in tissue regeneration. Methods Mol. Biol..

[42] Islam M.S., Ang B.C., Andriyana A., Afifi A.M. (2019). A review on fabrication of nanofibers via electrospinning and their applications. SN Appl. Sci..

[43] Marquez A.L., Gareis I.E., Dias F.J., Gerhard C., Lezcano M.F. (2022). Methods to Characterize Electrospun Scaffold Morphology: A Critical Review. Polymers.

[44] Fioretta E.S., Simonet M., Smits A.I.P.M., Baaijens F.P.T., Bouten C.V.C. (2014). Differential response of endothelial and endothelial colony forming cells on electrospun scaffolds with distinct microfiber diameters. Biomacromolecules.

[45] Ko Y.-G., Park J.H., Lee J.B., Oh H.H., Park W.H., Cho D., Kwon O.H. (2016). Growth behavior of endothelial cells according to electrospun poly(D,L-lactic-co-glycolic acid) fiber diameter as a tissue engineering scaffold. Tissue Eng. Regen. Med..

[46] Milleret V., Hefti T., Hall H., Vogel V., Eberli D. (2012). Influence of the fiber diameter and surface roughness of electrospun vascular grafts on blood activation. Acta Biomater..

[47] Ahmed M., Ramos T., Wieringa P., van Blitterswijk C., de Boer J., Moroni L. (2018). Geometric constraints of endothelial cell migration on electrospun fibres. Sci. Rep..

[48] Yu C., Xing M., Wang L., Guan G. (2020). Effects of aligned electrospun fibers with different diameters on hemocompatibility, cell behaviors and inflammation in vitro. Biomed. Mater..

[49] Whited B.M., Rylander M.N. (2014). The influence of electrospun scaffold topography on endothelial cell morphology, alignment, and adhesion in response to fluid flow. Biotechnol. Bioeng..

[50] Li X., Wang X., Yao D., Jiang J., Guo X., Gao Y., Li Q., Shen C. (2018). Effects of aligned and random fibers with different diameter on cell behaviors. Colloids Surf. B Biointerfaces.

[51] Montero R.B., Vial X., Nguyen D.T., Farhand S., Reardon M., Pham S.M., Tsechpenakis G., Andreopoulos F.M. (2012). bFGF-containing electrospun gelatin scaffolds with controlled nano-architectural features for directed angiogenesis. Acta Biomater..

[52] Ghosh L.D., Jain A., Sundaresan N.R., Chatterjee K. (2018). Elucidating molecular events underlying topography mediated cardiomyogenesis of stem cells on 3D nanofibrous scaffolds. Mater. Sci. Eng. C Mater. Biol. Appl..

[53] Rüder C., Sauter T., Kratz K., Haase T., Peter J., Jung F., Lendlein A., Zohlnhöfer D. (2013). Influence of fibre diameter and orientation of electrospun copolyetheresterurethanes on smooth muscle and endothelial cell behaviour. Clin. Hemorheol. Microcirc..

[54] Kai D., Prabhakaran M.P., Jin G., Ramakrishna S. (2011). Guided orientation of cardiomyocytes on electrospun aligned nanofibers for cardiac tissue engineering. J. Biomed. Mater. Res. Part B Appl. Biomater..

[55] Huang C.-Y., Hu K.-H., Wei Z.-H. (2016). Comparison of cell behavior on pva/pva-gelatin electrospun nanofibers with random and aligned configuration. Sci. Rep..

[56] Mertgen A.-S., Yazgan G., Guex A.G., Fortunato G., Müller E., Huber L., Schneider R., Brunelli M., Rossi R.M., Maniura-Weber K. (2018). Controlling the surface structure of electrospun fibers: Effect on endothelial cells and blood coagulation. Biointerphases.

[57] Wang Z., Cui Y., Wang J., Yang X., Wu Y., Wang K., Gao X., Li D., Li Y., Zheng X.-L. (2014). The effect of thick fibers and large pores of electrospun poly(ε-caprolactone) vascular grafts on macrophage polarization and arterial regeneration. Biomaterials.

[58] Garg K., Pullen N.A., Oskeritzian C.A., Ryan J.J., Bowlin G.L. (2013). Macrophage functional polarization (M1/M2) in response to varying fiber and pore dimensions of electrospun scaffolds. Biomaterials.

[59] Li W.J., Laurencin C.T., Caterson E.J., Tuan R.S., Ko F.K. (2002). Electrospun nanofibrous structure: A novel scaffold for tissue engineering. J. Biomed. Mater. Res..

[60] Soliman S., Sant S., Nichol J.W., Khabiry M., Traversa E., Khademhosseini A. (2011). Controlling the porosity of fibrous scaffolds by modulating the fiber diameter and packing density. J. Biomed. Mater. Res. A.

[61] De Valence S., Tille J.-C., Giliberto J.-P., Mrowczynski W., Gurny R., Walpoth B.H., Möller M. (2012). Advantages of bilayered vascular grafts for surgical applicability and tissue regeneration. Acta Biomater..

[62] Bergmeister H., Schreiber C., Grasl C., Walter I., Plasenzotti R., Stoiber M., Bernhard D., Schima H. (2013). Healing characteristics of electrospun polyurethane grafts with various porosities. Acta Biomater..

[63] Heath D.E., Lannutti J.J., Cooper S.L. (2010). Electrospun scaffold topography affects endothelial cell proliferation, metabolic activity, and morphology. J. Biomed. Mater. Res. A.

[64] Kim H.H., Kim M.J., Ryu S.J., Ki C.S., Park Y.H. (2016). Effect of fiber diameter on surface morphology, mechanical property, and cell behavior of electrospun poly(ε-caprolactone) mat. Fibers Polym..

[65] Xu C., Yang F., Wang S., Ramakrishna S. (2004). In vitro study of human vascular endothelial cell function on materials with various surface roughness. J. Biomed. Mater. Res. A.

[66] Blann A.D., Woywodt A., Bertolini F., Bull T.M., Buyon J.P., Clancy R.M., Haubitz M., Hebbel R.P., Lip G.Y.H., Mancuso P. (2005). Circulating endothelial cells. Biomarker of vascular disease. Thromb. Haemost..

[67] Li H., Xu Y., Xu H., Chang J. (2014). Electrospun membranes: Control of the structure and structure related applications in tissue regeneration and drug delivery. J. Mater. Chem. B.

[68] Lowery J.L., Datta N., Rutledge G.C. (2010). Effect of fiber diameter, pore size and seeding method on growth of human dermal fibroblasts in electrospun poly(epsilon-caprolactone) fibrous mats. Biomaterials.

[69] Haider A., Haider S., Kang I.-K. (2018). A comprehensive review summarizing the effect of electrospinning parameters and potential applications of nanofibers in biomedical and biotechnology. Arab. J. Chem..

[70] Chung T.-W., Liu D.-Z., Wang S.-Y., Wang S.-S. (2003). Enhancement of the growth of human endothelial cells by surface roughness at nanometer scale. Biomaterials.

[71] Giol E.D., van Vlierberghe S., Unger R.E., Schaubroeck D., Ottevaere H., Thienpont H., Kirkpatrick C.J., Dubruel P. (2018). Endothelialization and Anticoagulation Potential of Surface-Modified PET Intended for Vascular Applications. Macromol. Biosci..

[72] Xu C. (2004). Aligned biodegradable nanofibrous structure: A potential scaffold for blood vessel engineering. Biomaterials.

[73] Kämmerer P.W., Gabriel M., Al-Nawas B., Scholz T., Kirchmaier C.M., Klein M.O. (2012). Early implant healing: Promotion of platelet activation and cytokine release by topographical, chemical and biomimetical titanium surface modifications in vitro. Clin. Oral Implant. Res..

[74] Koh L.B., Rodriguez I., Venkatraman S.S. (2010). The effect of topography of polymer surfaces on platelet adhesion. Biomaterials.

[75] Chen H., Song W., Zhou F., Wu Z., Huang H., Zhang J., Lin Q., Yang B. (2009). The effect of surface microtopography of poly(dimethylsiloxane) on protein adsorption, platelet and cell adhesion. Colloids Surf. B Biointerfaces.

[76] Lutter C., Nothhaft M., Rzany A., Garlichs C.D., Cicha I. (2015). Effect of specific surface microstructures on substrate endothelialisation and thrombogenicity: Importance for stent design. Clin. Hemorheol. Microcirc..

[77] Minelli C., Kikuta A., Tsud N., Ball M.D., Yamamoto A. (2008). A micro-fluidic study of whole blood behaviour on PMMA topographical nanostructures. J. Nanobiotechnology.

